# The effect of nurses’ perceived social support on turnover intention: the chain mediation of occupational coping self-efficacy and depression

**DOI:** 10.3389/fpubh.2025.1527205

**Published:** 2025-03-27

**Authors:** Zhenfan Liu, Xiaoting Yan, Guifang Xie, Jing Lu, Zhitong Wang, Cui Chen, Jijun Wu, Wei Qing

**Affiliations:** ^1^Department of Nursing, Deyang People’s Hospital, Deyang, China; ^2^Department of Nursing, Sichuan Nursing Vocational College, Chengdu, China; ^3^Department of Nursing, Mianzhu Second People’s Hospital, Mianzhu, China

**Keywords:** nurses, perceived social support, occupational coping self-efficacy, depression, turnover intention, chain mediating effects

## Abstract

**Objective:**

To explore the chain mediating role of occupational coping self-efficacy and depression in the mechanism of nurses’ perceived social support on turnover intention.

**Methods:**

A convenience sample of 390 nurses from five general hospitals was surveyed from April–June 2024 using the General Information Questionnaire, the perceived social support scale, the occupational coping self-efficacy scale, the depression scale, and the turnover intention scale to construct and validate the chain mediated effects model.

**Results:**

The results of this study showed that nurses’ turnover intention scored (13.38 ± 4.83), perceived social support scored (70.25 ± 11.55), occupational coping self-efficacy scored (37.22 ± 5.45), and depression scored 6 (3.00, 11.00). The direct effect of perceived social support on nurses’ turnover intention their jobs was significant with an effect value of −0.1793; occupational coping self-efficacy and depression as separate mediating effects and chain mediating effects of both were − 0.0281, −0.0343, and − 0.0474, respectively.

**Conclusion:**

Nurses’ turnover intention is at a high level, and the chain-mediated effects of occupational coping self-efficacy and depression in the mechanism of nurses’ perceived social support on their turnover intention are established. Managers should pay attention to nurses with high turnover intention, increase their level of perceived social support, promote their occupational coping self-efficacy, and reduce their depression to further reduce their turnover intention and enable them to actively engage in their work.

## 1 Introduction

It is well known that nurses are the backbone of the global healthcare industry and play a very important role in promoting human health. However, with the aging of the global population, the outbreak of infectious diseases, and the changing spectrum of diseases, the work tasks and workloads undertaken by nurses are also deepening (1). At the same time, the social status and economic income of nurses do not match their work tasks, and the value of their work is not reflected accordingly, with a high percentage of nurses showing a turnover intention (2). Turnover intention mainly refers to the idea or willingness of employees to leave the organization after experiencing dissatisfaction in the organization, which will directly predict the departure behavior of nurses (3). One study showed that 22.02% of nurses have frequent thoughts of changing jobs, and as many as 70.80% of nurses will choose to leave their jobs in the next year due to dissatisfaction with their current job (4). By 2025, the United States will also have close to 260,000 fewer nurses (5). This shows that the shortage of nurses has become an intractable problem globally. When nurses show serious turnover intention, it will not only affect the recruitment cost and talent training cost of hospitals, but also directly affect the supply of nursing services (6, 7). Therefore, it is necessary to reduce the willingness of nurses to leave.

Nurses’ turnover intention their jobs is influenced by a variety of factors, and perceived social support is one of the important influences (35). Perceived social support, as an effective external resource, reflects the degree to which individuals can perceive and actually obtain external help. Research has shown that effective social support can help nurses better cope with difficult situations and positively cope with work stress, thus reducing negative emotions and enhancing work motivation (8, 36). In addition, a survey of emergency department nurses’ turnover intention their jobs found that when individuals perceived more social support, their job satisfaction increased significantly, burnout was alleviated, and their willingness to leave decreased (9). These studies have confirmed that perceived social support has a positive effect on turnover intention, but the specific mechanism of action has not been fully elaborated.

Occupational coping self-efficacy refers to the subjective evaluation and awareness that individuals can effectively cope with and complete their own work, and it is an effective judgment of their own behavioral ability and psychological state. It has been found that the higher the sense of occupational coping self-efficacy, the more nurses can show positive engagement in their work, and higher work engagement can directly reduce the turnover rate of nurses (10). Meanwhile, occupational coping self-efficacy, as an important role factor of burnout, can effectively reduce nurses’ boredom with their careers, and burnout has a direct contributing effect on the turnover intention the job (11). In summary, it is reasonable to believe that nurses’ occupational coping self-efficacy has a positive contributing effect on reducing turnover intention. In addition, according to the resource exchange theory, when nurses perceive external support for their work, their self-efficacy and coping ability can be further enhanced, thus showing positive motivation, which in turn reduces their turnover intention. Considering the above, the mediating role between perceived social support and turnover intention. Hypothesis 1: Self-efficacy in occupational coping mediates the relationship between perceived social support and turnover intention.

Depression may be another variable that comprehends the existence of an influential role between social support and turnover intention. As a negative emotional experience, depression can directly affect the level of mental health of nurses (12). When nurses are under more pressure, they can develop negative burnout toward their work and even the idea of quitting. And organizational support has a direct negative predictive effect on depression (13, 14). When nurses perceive more support, they are bound to promote the construction of positive emotional experiences, effectively reduce negative psychological emotions, and engage in their work in a positive state. This led to Hypothesis 2: Depression mediates the relationship between perceived social support and turnover intention. Meanwhile, according to the theory of individual-environmental matching, the factors that cause individual stress include the interaction between the individual and the environment, in addition to the individual’s intrinsic (self-efficacy, etc.) or extrinsic environmental (sense of social support, etc.) factors (15). Relevant studies have also confirmed that social support promotes the development of self-efficacy and has a negative predictive effect on psychological mood, thus reducing the occurrence of depression in individuals and mitigating the turnover rate of nurses (16). This led to Hypothesis 3: Occupational coping self-efficacy and depression act as chain mediators between apprehension of social support and turnover intention.

In summary, the interaction between nurses’ positive psychological resources and negative experiences has received extensive attention in the field of nursing, but fewer reports have elaborated on the mechanisms of nurses’ occupational coping self-efficacy, navigational social support, depression, and turnover intention. While analyzing the correlation of the four variables, this study aimed to explore the direct effect of perceived social support on turnover intention and the mediating role played by occupational coping self-efficacy and depression, with the aim of alleviating nurses’ depression and reducing the turnover rate of nurses, so as to provide a reference basis for improving the quality of nursing care and ensuring patient safety.

## 2 Methods

### 2.1 Participants

In this study, a cross-sectional survey was conducted in April–June (17), and 390 nurses from 5 general hospitals were selected as survey respondents by convenience sampling. Inclusion criteria: (1) having obtained the Chinese nurse practitioner’s qualification certificate and having worked in nursing for ≥3 months; (2) knowing the content of this study and willing to participate in the survey; Exclusion criteria: nurses on vacation or in training during the survey period.

Sample size formula, before the formal survey we first conducted a pre-survey, calculated the standard deviation of nurses’ turnover intention score σ is 5.25, so set σ = 5.25, δ=1, α=0.05, = 1.96 (18). The prognosis yielded *n* = 106, and considering 10 to 20% invalid questionnaires, the sample size obtained was calculated to be at least 117 to 128, and the actual sample included in this study was 390. The study was approved by the Ethics Committee (No. 2023–04-070-K01).

### 2.2 Methodology

#### 2.2.1 Survey instruments

##### 2.2.1.1 General information questionnaire

Determined by the research team after reviewing the literature and consisted of entries such as gender, age, hospital level, title, form of employment, and highest level of education of the nurses.

##### 2.2.1.2 Occupational coping self-efficacy scale

Compiled by Pisanti et al. and translated and revised by Chinese scholar Zhai Yanxue et al. (9), the scale has high reliability and validity (19, 20). The Cronbach′s *α* coefficient of the scale was 0.882. Ding Xing et al. found the Cronbach′s α coefficient of the scale to be 0.937 in a survey of nurses. The scale consisted of a relationship getting along difficulty dimension and a career burden dimension with 9 entries. The scale was scored on a 5-point Likert scale from “unable to cope easily” to “completely able to cope easily,” with a total score of 9 to 45, and the higher the score, the better the nurses’ self-efficacy for occupational coping. The Cronbach’s alpha coefficient for this scale in this study was 0.911.

##### 2.2.1.3 Perceived social support scale

Compiled by Zimet et al. and translated and revised by Chinese scholars Jiang Qianjin et al., the scale has high reliability and validity (21, 22). The Cronbach′s *α* coefficient of the scale was 0.990. Duan Xueyi et al. found that the Cronbach′s α coefficient of the scale was 0.976 in a survey of nurses (17). The scale consists of 3 dimensions of family support, friend support, and other support with a total of 12 entries. The scale was scored on a 7-point Likert scale from “strongly disagree to strongly agree” on a scale of 1–7, and the total score of the scale ranged from 12 to 84, with higher scores indicating that nurses perceived more social support. The Cronbach’s alpha coefficient for this scale in this study was 0.945.

##### 2.2.1.4 9-item patient health questionnaire scale, PHQ-9

Validated by Kroenke K et al. (23), the scale was widely used by Chinese scholars in surveys of nurses, and the scale has high reliability, The Cronbach′s *α* coefficient of the scale was 0.962 (1). The PHQ-9 was designed to assess the individual’s depressive symptoms over the past 2 weeks, with 9 questions on a 4-point scale ranging from 0 to 3, from “none” to “almost every day,” for a total scale score of 0 to 27. The scale scores ranged from 0 to 4 for no symptoms of depression, 5 to 9 for mild symptoms, 10 to 14 for moderate symptoms, 15 to 19 for moderate to severe symptoms, and 20 to 27 for severe symptoms. The Cronbach’s alpha coefficient for this scale in this study was 0.885.

##### 2.2.1.5 The turnover intention scale

The scale was compiled by Michaels and Spector et al. (24) and translated by Chinese scholars Li Dong Rong (25), and has been widely used to measure the level of turnover intention a job among Chinese nurses, and the scale has a high level of reliability, The Cronbach′s *α* coefficient of the scale was 0.841(3). The scale consisted of 3 dimensions, including turnover intention I: possibility of quitting (2 items), turnover intention II: motivation to find other jobs (2 items), and turnover intention III: possibility of obtaining other jobs (2 items), with a total of 6 items. The scale was based on a 4-point Likert scale, with “never” to “often” scored from 1 to 4, and the total score was 6 to 24. The higher the score, the stronger the nurses’ turnover intention. Judging criteria: The total mean score of ≤1, ≤2, ≤3, and > 3 divided the turnover intention into four grades, which represented very low, low, high, and very high turnover intention in that order. The Cronbach’s *α* coefficient for this scale in this study was 0.975.

#### 2.2.2 Survey methods

This study adopts a network APP online survey (Wen Juan Xing, wjx.cn). First, the project leader edited the questionnaire in accordance with the principles of questionnaire writing, including the purpose, content, significance, filling in matters, informed consent, and confidentiality of the questionnaire, and at the same time imported the questionnaire into the questionnaire star to create a link to conduct a pre-survey, and according to the results of the analysis of the pre-survey to further improve the content of the questionnaire to determine the final version of the survey; second, the project leader contacted the relevant liaison officers of the hospitals selected, and after obtaining their Secondly, the project leader contacted the relevant liaison officers of the selected hospitals and, after obtaining their informed consent, conducted survey training for all the liaison officers in the form of videoconferencing, and then sent the link and QR code of the questionnaire to the research subjects who met the criteria after passing the training; lastly, the research subjects read the relevant contents after receiving the link or the QR code, and completed the questionnaire after clicking on the button of agreeing to the survey. In order to ensure the validity and completeness of the questionnaire, all questionnaire entries are mandatory, and each IP address can only answer once, and cannot be submitted repeatedly. In order to ensure the confidentiality of the results of the study, the questionnaire does not show the name and the name of the hospital, and all the results of the study are coded and can only be viewed by the data analyst. A total of 402 questionnaires were collected, and after screening out the questionnaires with obvious errors in logic, and questionnaires with an answer time of <180 s or > 1800 s were regarded as invalid questionnaires, and 12 questionnaires were excluded, a total of 390 valid questionnaires were recovered, with an effective recovery rate of 97.0%.

#### 2.2.3 Statistical methods

The questionnaire results were directly exported from the backend of Questionnaire Star. SPSS 25.0 software was used for statistical analysis, normally distributed measures were expressed as mean ± standard deviation, skewed measures were expressed as M (P_25_, P_75_); count data were described by the number of people and percentage; the relationship between the four variables was explored using Spearman correlation analysis; mediation effects were analyzed using the SPSS macro program prepared by Hayes. PROCESS plug-in 6; Bootstrap method was used to calculate the 95% CI by repeated sampling 5,000 times, if none of the results contained 0, the mediation effect was significant. Differences were considered statistically significant at *p* < 0.05.

## 3 Results

### 3.1 General information about the nurses in this group

A total of 390 nurses were included in this study, including 33 (8.5%) male nurses and 357 (91.5%) female nurses; age:33.74 ± 7.35 years, 49 (12.6%) aged 21–25 years, 99 (25.4%) aged 26–30 years, 178 (45.6%) aged 31–40 years, and 64 (16.4%) >40 years; 261 (66.9%) in Level IIIA hospitals, 29 (7.4%) in Level IIIB hospitals, 100 (25.9%) in Level II hospitals; 196 (50.3%) in junior titles, 166 (42.6%) in intermediate titles, and 28 (7.1%) in senior titles; 79 (20.3%) on the staff, 311 (79.7%) on contract; 64 head nurses (16.4%), 326 general nurses (83.6%); 63 nurses with specialized education (16.2%), 308 nurses with bachelor’s degree (79.0%), 19 nurses with postgraduate education (4.8%).

### 3.2 Nurses’ turnover intention, perceived social support, depression and occupational coping self-efficacy scores in our group

The results of this study showed that the nurses’ turnover intention score was (13.38 ± 4.83), of which 226 (57.9%) had low turnover intention, 56 (14.4%) had high turnover intention, and 73 (18.7%) had very high turnover intention; the perceived social support score was (70.25 ± 11.55); the occupational coping self-efficacy score was (37.22 ± 5.45); the depression score was 6 (3.00, 11.00), of which 241 (61.8%) were screened for symptoms of depression, of which 129 (33.1%) had mild symptoms. 5.45); depression score was 6 (3.00, 11.00), of which 241 (61.8%) were screened for depressive symptoms, of which 129 (33.1%) were mild, 69 (17.7%) were moderate, 41 (10.5%) were moderately severe and 2 (0.5%) were severe.

### 3.3 Correlation of nurses’ turnover intention, perceived social support, depression and occupational coping self-efficacy

The results of the current study showed that the nurses’ perceived social support score was positively correlated with the occupational coping self-efficacy score (*r* = 0.590, *p* < 0.01) and negatively correlated with the depression and turnover intention scores (*r* = −0.576, −0.662, *p* < 0.01); the occupational coping self-efficacy score was negatively correlated with the depression and turnover intention scores (*r* = − 0.668, −0.586, *p* < 0.01); depression score was positively correlated with the turnover intention score (*r* = 0.671, *p* < 0.01). See Table 1.

**Table 1:** Correlation of turnover intention, perceived social support, depression and occupational coping self-efficacy among nurses in this group (*n*, *r*).

Sports event	Turnover intention	Turnover intention I	Turnover intention II	Turnover intention III	Occupational coping self-efficacy	Professional burden	Difficulty getting along in relationships	Despondent	PERCEIVED social support	Family support	Friends Support	Support from others
Turnover intention	1.000											
Turnover intention I	0.967^**^	1.000										
Turnover intention II	0.973^**^	0.938^**^	1.000									
Turnover intention III	0.970^**^	0.940^**^	0.949^**^	1.000								
Occupational coping self-efficacy	−0.586^**^	−0.560^**^	−0.569^**^	−0.574^**^	1.000							
Occupational Burden Dimension	−0.566^**^	−0.545^**^	−0.550^**^	−0.553^**^	0.967^**^	1.000						
Difficulty getting along in relationships	−0.518^**^	−0.493^**^	−0.503^**^	−0.513^**^	0.882^**^	0.752^**^	1.000					
Despondent	0.671^**^	0.656^**^	0.666^**^	0.650^**^	−0.668^**^	−0.618^**^	−0.641^**^	1.000				
Perceived social support	−0.662^**^	−0.652^**^	−0.641^**^	−0.668^**^	0.590^**^	0.566^**^	0.539^**^	−0.576^**^	1.000			
Family support	−0.640^**^	−0.630^**^	−0.619^**^	−0.660^**^	0.563^**^	0.545^**^	0.518^**^	−0.526^**^	0.923^**^	1.000		
Friends Support	−0.598^**^	−0.589^**^	−0.579^**^	−0.599^**^	0.527^**^	0.508^**^	0.474^**^	−0.526^**^	0.937^**^	0.801^**^	1.000	
Support from others	−0.629^**^	−0.617^**^	−0.613^**^	−0.631^**^	0.574^**^	0.547^**^	0.530^**^	−0.573^**^	0.964^**^	0.855^**^	0.892^**^	1.000

^**^p < 0.01.

### 3.4 Chain mediating role of vocational coping self-efficacy, depression between perceived social support and turnover intention in this group of nurses

After standardizing the statistical data, using perceived social support as the independent variable, occupational coping self-efficacy, depression as the mediator variable, and turnover intention the job as the dependent variable, the PROCESS plug-in developed by Hayes was used, and model 6 was selected for the mediation effect test, and it was found that: perceived social support can significantly negatively predict turnover intention the job; perceived social support can significantly positively predict occupational coping self-efficacy and significantly negatively predicted depression; occupational coping self-efficacy can significantly negatively predict depression and turnover intention; and depression can significantly positively predict turnover intention. See Table 2.

**Table 2:** Regression model of the chain mediation model of vocational coping self-efficacy, depression between perceived social support and turnover intention in this group of nurses.

Regression equation		Fitness index			Significance of regression coefficients	
Outcome variable	Predictor variable	*R*	*R^2^*	*F*	*β*	*t*
Occupational coping self-efficacy	Perceived social support	0.503	0.254	131.728	0.238	11.477**
Depression	Perceived social support	0.701	0.491	186.595	−0.149	−7.857**
	Occupational coping self-efficacy				−0.455	−11.293**
Turnover intention	Perceived social support	0.776	0.603	195.286	−0.179	−3.110**
	Occupational coping self-efficacy				−0.118	7.643**
	Depression				0.317	−10.719**

**p < 0.01.

**Figure 1:**
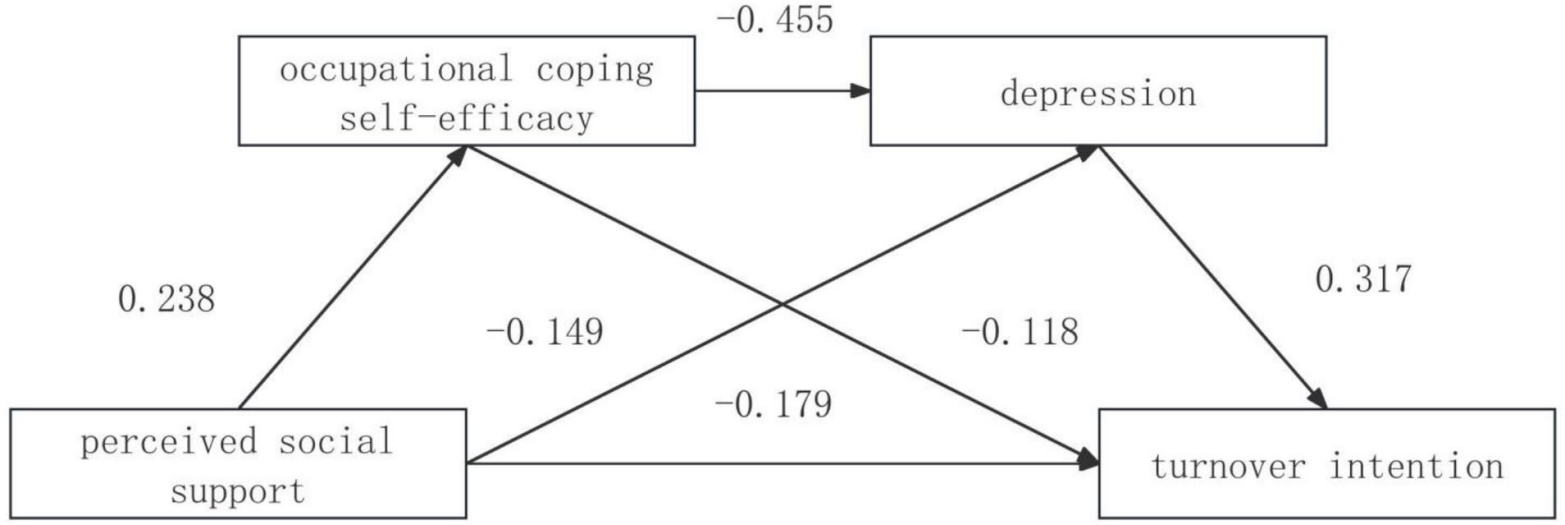
Chain mediation model of occupational coping self-efficacy, depression between perceived social support and turnover intention among nurses in this group.

The mediation effect was examined using Bootstrap method with 5,000 repeated samples, and the mediation model is shown in Figure 1. The results of the study found that the confidence interval of Path 1: perceived social support→occupational coping self-efficacy→turnover intention did not contain 0, i.e., the mediation effect of occupational coping self-efficacy between perceived social support and turnover intention was significant, and the amount of mediation effect was −0.0280; Path 2: The confidence interval of perceived social support→depression→turnover intention did not contain 0, i.e., the mediation effect of depression between perceived social support and turnover intention was significant, and the mediation effect was −0.0343; Path 3: The confidence interval of perceived social support→occupational coping self-efficacy→depression→turnover intention did not contain 0, i.e., the mediation effect of the chain between occupational coping self-efficacy and depression between perceived social support and turnover intention was significant, and the mediation effect was −0.0343. In addition, the confidence interval of the pathway: cognitive social support→cognitive self-efficacy→depression→turnover intention did not contain 0, which indicated that the direct effect of cognitive social support on turnover intention was still significant after the introduction of occupational coping self-efficacy and depression, and the direct effect was −0.1793, as shown in Table 3.

**Table 3:** Analysis of the mediating effects of occupational coping self-efficacy, depression in the relationship between nurses’ perceived social support and turnover intention the profession.

Trails	Path effects of standardization	Amount of effect/%	95% CI	
			Lower limit	Limit
perceived social support → occupational coping self-efficacy → turnover intention	−0.0281	9.7	−0.0550	−0.0014
perceived social support → depression → turnover intention	−0.0343	11.9	−0.0516	−0.0206
Appreciation social support → occupational coping self-efficacy → depression → turnover intention	−0.0474	16.4	−0.0726	−0.0289
Aggregate intermediary effect	−0.1098	38.0	−0.1516	−0.0773
Direct effect	−0.1793	62.0	−0.2248	−0.1338
Aggregate effect	−0.2891	100.00	−0.3155	−0.2628

## 4 Discussion

### 4.1 Nurses in this group have a high level of turnover intention and depressive symptoms, and perceived social support and occupational coping self-efficacy are at a moderate to high level

The study findings revealed that nurses’ turnover intention score was 13.38 ± 4.83, indicating a relatively high level, which aligns with Liu et al.’s survey results (9) but slightly lower than the score of 15.50 ± 3.44 observed in Jiangsu Province (6). This discrepancy may be attributed to regional differences. The current survey was conducted in western China, where compared to economically developed regions like Jiangsu Province, there exists a relatively scarce nursing workforce, more conservative cultural perceptions, and lower salary levels. Nurses in this region not only endure frequent night shifts but also face significant nurse–patient and interprofessional communication pressures. Moreover, their professional value often lacks recognition from families, colleagues, and society, collectively contributing to heightened turnover intention. Additionally, while 83.8% of surveyed nurses held bachelor’s degrees or higher, only 20.3% were on the staff nurses. In China’s healthcare system, on the staff nurses generally enjoy greater job stability, better welfare benefits, and clearer career progression compared to contract-based nurses. The inequitable treatment between these two groups may drive contract nurses to seek alternative employment opportunities, thereby exacerbating turnover intention (26).

The results of this study found that the depression score of nurses was 6 (3.00, 11.00), in which the incidence of depressive symptoms was 61.8%, which was higher than that of related studies at home and abroad (16, 27), suggesting that clinical nurses have serious mental health problems. This may be related to the different characteristics of the survey respondents. Among them, this survey also found that 28.7% of the nurses had moderate to severe depressive symptoms. This may be due to the fact that (1) 38.0% of the nurses in this study were under 30 years of age, and the lower the age, the more difficult it is for nurses to effectively cope with emergencies in the workplace due to a relative lack of clinical work experience and a relatively low level of psychological endurance, which results in a higher risk of depression; (2) with the increasing competition, the nurse population not only needs to fulfill their clinical work tasks, but also needs to (2) With increasing competition, nurses not only need to fulfill their clinical tasks, but also need to actively participate in various competitions and additional tasks such as writing scientific research papers and research projects, thus enduring greater work pressure, which leads to the emergence of depressive symptoms easily.

The results of the current study found that the nurses’ perceived social support score was (70.25 ± 11.55), which was at a moderately high level compared to the middle score of the scale, 48, and was higher than the results of the study by He Hong et al. (28). This may be due to the fact that (1) as the educational level of nursing managers continues to rise, management awareness and management ability are also tending to the direction of scientific and humane development, which enables clinical nurses to perceive more care and support from the organization, leadership and colleagues; (2) 91.5% of the nurses in this survey were female, and relative to male nurses, female nurses are more emotionally sensitive and better able to perceive others’ care and support for themselves; (3) 91.5% of the nurses in this survey were female, and compared to male nurses, female nurses were more emotionally sensitive and better able to perceive others’ care and support for themselves. Female nurses are more sensitive emotionally than male nurses, and are more able to perceive others’ concern and support for themselves.

The results of the current study found that the nurses’ occupational coping self-efficacy score was (37.22 ± 5.45), which was in the middle of the range compared with the scale’s median score of 27, higher than that of Wu Jijun et al.’s survey of ICU nurses (29). This may be related to the difference in the selection of the study population; compared with the nurses in ICU, the nurses in the general department were faced with a relatively low level of patients’ conditions, which made them bear a relatively low level of work pressure, and thus had the confidence to fulfill the corresponding work tasks.

### 4.2 Occupational coping self-efficacy mediates the relationship between nurses’ perceptions of social support and turnover intention

The results of the current study showed that occupational coping self-efficacy mediated the effect between nurses’ perceived social support and turnover intention, with a mediation effect value of −0.0281, accounting for 9.7% of the total effect. The mediating effect was −0.0281, which accounted for 9.9% of the total effect. This indicates that perceived social support not only directly affects nurses’ turnover intention, but also indirectly through the mediating effect of occupational coping self-efficacy. Perceived social support as an effective social resource, when nurses perceive more care and support from colleagues, organizations, society and family, the corresponding level of psychological elasticity will be effectively enhanced, and they will be able to effectively overcome the difficulties and pressures of work and be hopeful about the future development of their own careers; in addition, occupational coping self-efficacy, as a protective factor for the turnover intention, will further increase the psychological resilience of nurses when their intrinsic motivation and confidence are fully satisfied. In addition, occupational coping self-efficacy, as a protective factor for the turnover intention, when the internal motivation and confidence of individuals are fully satisfied, it will further motivate the nurses to take a positive state to devote themselves to their work and continuously pursue a higher performance, thus effectively reducing the need and motivation of nurses to escape from the hospital workplace, and making the rate of separation decrease (30).

### 4.3 The mediating role of depression between nurses’ perceived social support and their turnover intention

The results of the current study showed that depression mediated the effect between nurses’ perceived social support and turnover intention, with a mediating effect value of −0.0343, accounting for 11.9% of the total effect. It shows that perceived social support can not only directly affect nurses’ turnover intention, but also indirectly through the mediating effect of depression. When nurses bear more pressure from work, it will directly consume their emotional resources, making their emotional regulation ability decrease, which leads to the occurrence of depressive symptoms. And nurses who are troubled by negative psychological emotions for a long time will develop the idea of fleeing from the workplace, which aggravates the occurrence of separation behavior. However, comprehending social support has a protective effect on negative psychology such as depression, which is directly related to an individual’s mental health (31, 32). When nurses perceive more support and care from the outside world, it directly reduces their emotional exhaustion, counteracts negative mental health levels, and allows them to engage in their work with enthusiasm, resulting in a decreased turnover intention.

### 4.4 Mediating role of occupational coping self-efficacy and depression in nurses’ perceived social support and turnover intention

The results of the current study showed that occupational coping self-efficacy and depression had a chain-mediated effect between nurses’ perceived social support and turnover intention, with a chain-mediated effect value of −0.0474, accounting for 16.4% of the total effect. This suggests that perceived social support not only directly affects nurses’ turnover intention, but also indirectly through the mediating effect of depression. Navigating social support can promote the establishment of an individual’s internal self-efficacy, so that he or she can show self-confidence and positivity in the workplace. And individuals with higher occupational coping self-efficacy showed higher levels of resilience, were able to overcome the negative psychology caused by work stress, maintained positive work beliefs, effectively reduced occupational stress, and enhanced job satisfaction, so that they would not easily choose to leave their current jobs (4, 33). On the other hand, when nurses’ occupational stressors are excessive and negative distress arises, higher levels of perceived social support enhance nurses’ utilization of support resources, enabling them to access positive emotional experiences, reduce negative disturbances, and rationally cope with stress, thus further reducing the emergence of the turnover intention.

## 5 Limitations

This study also has some limitations due to time and effort constraints. First, this study took online questionnaire filling, and the results presented are somewhat subjective. Second, most of the samples in this study were from the same region, and the results presented are not representative enough. Also, this study did not explore the willingness of nurses to leave their jobs in different hospital levels and different departments. Finally, this study was only a cross-sectional survey, and the causal inference of the hypothesized relationship between the relevant variables was somewhat limited. In the future, we need to further expand the sample scope and sample size to make the sample more representative; colleagues, we need to further explore the effects of different hospital levels and different departments on nurses’ depressive symptoms and willingness to leave; finally, we need to conduct a longitudinal study to observe the trend of the variables, so as to provide reference bases for the subsequent development of targeted interventions to reduce the incidence of nurses’ depressive symptoms and their willingness to leave. We also need to conduct a longitudinal study to observe the trend of change among variables, so as to provide a reference basis for the subsequent development of targeted interventions to reduce the incidence of nurses’ depressive symptoms and their willingness to leave.

## 6 Conclusion

Nurses’ turnover intention is at a high level, and the chain mediating role of occupational coping self-efficacy and depression between nurses’ perceived social support and turnover intention is established. Nursing managers were prompted to: (1) help nurses establish a social support network system in which nurses can exchange experiences and acquire stress coping strategies; (2) actively publicize the professional value of nurses, so that nurses can acquire more recognition and support from colleagues, society, and families, and awaken nurses’ motivation for their work; (3) pay attention to nurses’ professional coping self-efficacy dynamics and its improvement, and timely understand their performance in dealing with emergencies. Enhancement, timely understanding of the needs and difficulties in dealing with emergencies, and actively carry out internal efficacy enhancement training courses; (4) pay attention to the negative psychological level of nurses, and carry out targeted intervention and training work for nurses with different characteristics, such as positive thinking intervention therapy and multimodal anti-stress training. From the external social support and internal self-efficacy 2 aspects of multi-dimensional to maintain the positive psycho-emotional experience of nurses, thereby reducing the turnover intention, to maintain the stability and development of the nursing team (34).

## Data Availability

The raw data supporting the conclusions of this article will be made available by the authors, without undue reservation.
